# Low N Fertilizer Application and Intercropping Increases N Concentration in Pea (*Pisum sativum* L.) Grains

**DOI:** 10.3389/fpls.2018.01763

**Published:** 2018-11-30

**Authors:** Falong Hu, Yan Tan, Aizhong Yu, Cai Zhao, Jeffrey A. Coulter, Zhilong Fan, Wen Yin, Hong Fan, Qiang Chai

**Affiliations:** ^1^Gansu Provincial Key Laboratory of Aridland Crop Science, Lanzhou, China; ^2^College of Agronomy, Gansu Agricultural University, Lanzhou, China; ^3^College of Forestry, Gansu Agricultural University, Lanzhou, China; ^4^Department of Agronomy and Plant Genetics, University of Minnesota, St. Paul, MN, United States

**Keywords:** N fertilizer management, intercropping, pea, N translocation, nitrogen harvest index

## Abstract

Sustainable intensification of pulses needs reduced input of nitrogen (N) fertilizer with enhanced crop nutritional quality and yield. Therefore, increasing N harvest in grains (sink organs) by improving N remobilization is of key importance. Previous research has shown that a lower dose of N fertilizer effectively increases the rate of N remobilization, while intercropping improves the grain N concentration in pea (*Pisum sativum* L.). However, it is unknown whether intercropping can facilitate this N fertilizer effect to increase N remobilization, and thereby enhance the N harvest index (NHI). In this study, we determined N allocation among different organs of pea plants, N translocation from leaf and stem tissues to pods, N_2_ fixation, N utilization efficiency, and NHI of pea plants grown alone or intercropped with maize (*Zea mays* L.) with different N fertilization treatments in a field experiment in northwestern China from 2012 to 2014. A base application of 90 kg N ha^−1^ at sowing and top-dress application of 45 kg N ha^−1^ at flowering integrated with maize–pea intercropping increased N allocation to pod tissues, N translocation to grains, and NHI of pea plants. Compared with the application of 90 kg N ha^−1^ at sowing and 135 kg N ha^−1^ top-dressed at flowering, reducing the top-dress application of N fertilizer to 45 kg N ha^−1^ increased N allocation to intercropped pea plants by 8%. Similarly, N translocation to grains from leaf and stem tissues was increased by 37.9 and 43.2%, respectively, enhancing the NHI by 40.1%. A positive correlation between N_2_ fixation and NHI was observed, implying that N_2_ fixation improves N concentration in grain sinks. Thus, our data show that growing pulses in an intercropping system with reduced N fertilization are essential for maximizing N translocation, improving nutritional quality, and preventing the loss of N through leaching, thereby avoiding potential groundwater contamination.

## Introduction

Nitrogen (N) is a vital element for adequate crop growth and the production of grains, and supplemental N fertilizer is often required to optimize crop yield (IFA, 2009; Kaur et al., 2017). In China, N fertilizer application has increased the grain yield of crops from 83.4 to 474.2 Mt from 1961 to 2009 (Fan et al., 2012), thus making it possible to feed one-fifth of the world population from < 9% of the world arable land (Jiao et al., 2016). However, N fertilizer is frequently applied in excess of crop requirements (Pearman et al., 1977), which has led to serious environmental problems such as impaired water quality, degraded soils, and increased greenhouse gas emissions (Cameron et al., 2013). To maintain crop yield while reducing environmental risks, it is necessary to improve the N use efficiency of crop plants and reduce N input (Brooker et al., 2016). To achieve these goals, it is important to enhance the remobilization efficiency of N in crops (Schjoerring et al., 1995; Masclaux-Daubresse et al., 2010).

Senescence of vegetative organs is critical during the reproductive growth phase of crops, as it allows the translocation of nutrients from non-reproductive organs to grains (Dreccer et al., 2000). The process of N remobilization is crucial for increasing crop yield and reducing N loss through senesced organs (Masclaux-Daubresse et al., 2010). Remobilization of N from vegetative to reproductive organs is affected by the genotype and environment, and is favored when soil nitrate availability constrains crop growth (Lemaître et al., 2008). Studies have shown that N deficiency, an abiotic stress, triggers earlier, and more rapid remobilization of N (Schjoerring et al., 1995; Mu et al., 2016). This contributes to higher grain protein content and seed nutritional quality (Samonte et al., 2006; Klimek-Kopyra et al., 2018a). However, grain protein content is negatively correlated with yield; thus, it is important to identify approaches that increase N harvest index (NHI) while maintaining yield (Masclaux-Daubresse et al., 2010).

Intercropping is the practice of growing two or more crops simultaneously in a field, and is being increasingly adopted as a sustainable farming practice (Lithourgidis et al., 2011a; Klimek-Kopyra et al., 2018b). Because of the improved water, nutrient, and radiation use efficiency, intercropping systems produce greater yield with lower environmental impact and economic risk compared with single cropping systems (Monzon et al., 2014). Additionally, intercropping effectively increases grain N content (Neugschwandtner and Kaulm, 2015), which is important for improving NHI. In oat (*Avena sativa* L.)–pea (*Pisum sativum* L.) intercropping, increased N content in peas has been attributed to enhanced N remobilization (Neumann et al., 2007). In wheat (*Triticum aestivum* L.)–pea intercropping, grain N content of both intercropped components is greater than that of the corresponding sole-cropped species (Ghaley et al., 2005; Klimek-Kopyra et al., 2018c). However, in barley (*Hordeum vulgare* L.)–lentil (*Lens culinaris* L.) intercropping, greater interspecific competition of barley restricted N allocation to lentil seed, which reduced grain N content (Schmidtke et al., 2004).

Pea is a popular protein-rich pulse crop suitable for animal feed and human consumption (Ghaley et al., 2005). It is commonly intercropped with forage and grain crops (Lithourgidis et al., 2011b). In northwestern China, pea is widely intercropped with maize (*Zea mays* L.) (Chai et al., 2013). This cropping system has demonstrated enhanced agronomic productivity, atmospheric N_2_ fixation, and reduced carbon emission (Chai et al., 2013; Hu et al., 2017). In this cropping system, pea is more competitive than maize (Hu et al., 2016), indicating the potential for increased N remobilization to grains in intercropped pea plants compared with sole-cropped pea. The objective of this study was to investigate whether intercropping is effective in increasing N remobilization in pea plants with reduced N fertilizer input. We hypothesized that pea–maize intercropping would improve the NHI of pea with reduced N fertilizer input by (i) enhancing N translocation and ii) increasing nitrogen utilization efficiency (NutE). To test this hypothesis, we determined (i) N content and allocation in leaf, stem, and pod tissues of pea, (ii) N translocation from leaf and stem tissues to pods, and (iii) N_2_ fixation, NutE, and NHI of sole and intercropped pea.

## Materials and methods

### Experimental site

**Figure 1:**
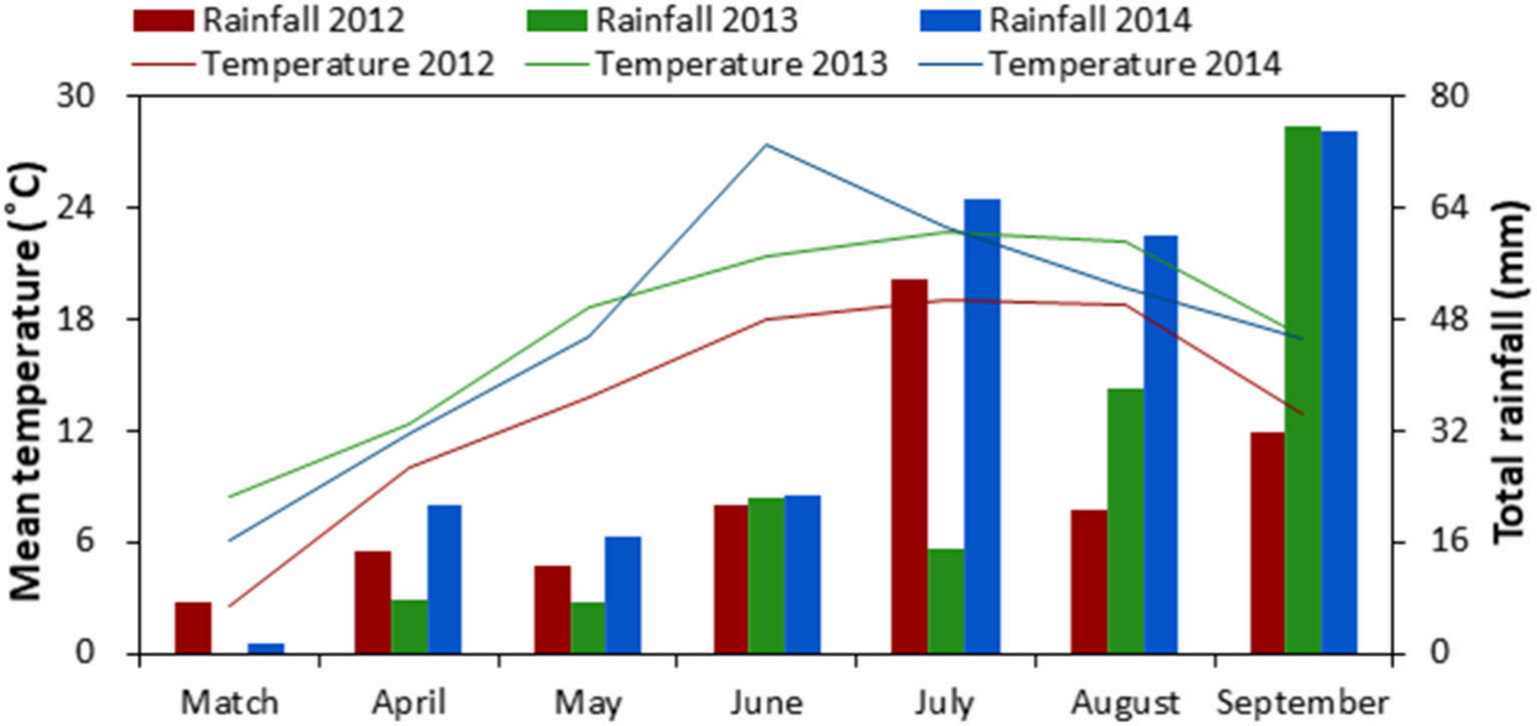
Monthly mean air temperature and total rainfall during the growing season of maize-pea intercropping in 2012, 2013 and 2014, northwestern China.

A field experiment was conducted at the Oasis Agricultural Experimental Station of Gansu Agricultural University (37°30′ N, 103°5′ E, 1776 m a.s.l.), located in the Hexi Oasis Irrigation District of Gansu Province in northwestern China from 2012 to 2014. This region accumulates abundant heat and light for one crop per year but insufficient for two crops a year. At this experimental site, long-term (1960–2009) average annual sunshine duration is 2,945 h, solar radiation is 5,670 J m^−2^, accumulated temperature (above 10°C) is 2,985°C, and mean annual air temperature is 7.2°C. Each year there was a 156-d frost-free period, 155 mm of precipitation, which mainly occurred during June to September (Figure 1), and at least 2,400 mm of evaporation. Soil at the experimental site is classified as an Aridisol (FAO/UNESCO, 1988). Prior to the experiment, soil properties of the top 0–30 cm soil layer were 8.0 pH (1:2.5 soil:water), 11.3 g kg^−1^ soil organic carbon, 1.44 g cm^−3^ soil bulk density, 0.94 g kg^−1^ total N, 29.2 mg kg^−1^ available phosphorous (P; Olsen-P), and 152.6 mg kg^−1^ available potassium (K; NH_4_OAc-extractable-K).

### Experimental design and crop management

The experimental design was a split-plot arrangement in randomized complete block design with three replications. Main plots were defined by the cropping system, and subplots were defined by N fertilization treatment. Two cropping systems were used: sole pea and pea intercropped with maize. The N fertilization treatments included four top-dressing methods (N0, N45, N90, and N135). The N0 treatment served as a non-N-fertilized control. In the N45, N90, and N135 treatments in sole pea, 45, 90, and 135 kg N ha^−1^ were applied as top-dress application at flowering, respectively, in addition to 90 kg N ha^−1^ applied as a base application at sowing (Table 1). In the intercropping system, N fertilizer was applied separately to pea and maize strips; intercropped pea received the same amount of base fertilizer per unit as sole pea, and intercropped maize received the same amount of N fertilizer as the corresponding component pea. All plots received a base application 150 kg P_2_O_5_ ha^−1^ at sowing using calcium superphosphate (0-16-0 of N-P_2_O_5_-K_2_O) in the N0 treatment, and diammonium phosphate (18-46-0 of N-P_2_O_5_-K_2_O) in the N45, N90, and N135 treatments. N fertilization in pea strips was performed by uniformly broadcasting urea on the soil surface, while N fertilization in maize strips was achieved by applying urea in a 3-cm diameter hole (10 cm deep), approximately 4–5 away cm from the maize stem, and subsequently sealing the hole with soil. Irrigation water was applied on the day after each N application to dissolve urea into the soil.

**Table 1:** Nitrogen (N) fertilization (kg N ha^−1^) for intercropped and sole pea by N fertilization treatment.

**N treatment^a^**	**Sole pea**			**Intercropped pea^b^**		
	**Base N**	**Top-dressed N**	**Total N**	**Base N**	**Top-dressed N**	**Total N**
N0	0	0	0	0	0	0
N45	90	45	135	38	19	57
N90	90	90	180	38	38	76
N135	90	135	225	38	57	95

a *N0 is the non-N fertilized control. N45, N90, and N135 represent N fertilizer applied at 90 kg N ha^−1^ as base fertilizer at sowing plus 45, 90, and 135 kg N ha^−1^ top-dressed at flowering, respectively*.

b *Intercropped pea received the same net area-based N fertilizer rate as corresponding sole pea*.

Pea cv. Long-wan No. 1 was sown on April 1, 2, and 1 and harvested on July 6, 4, and 10 in 2012, 2013, and 2014. Maize cv. Xian-yu 335 was sown on April 21, 22, and 25 and harvested on September 22, 25, and 29 in 2012, 2013, and 2014, respectively. Each plot was 45.6 m^2^ (5.7 × 8 m). In intercropping plots, a strip of four rows of pea with 20-cm row spacing alternated with a strip of three rows of maize with 40-cm row spacing, and each plot contained three pairs of pea–maize strips. In each intercropping plot, pea occupied 8/19th of the total area, while maize occupied 11/19th of the total area of the plot. Sole pea crop was established at 900,000 plants ha^−1^. The area-based plant population for intercropped pea (380,000 plants ha^−1^) was identical to that of sole-cropped pea, according to the area occupation ratio. Intercropped maize was established at 52,000 plants ha^−1^. Supplemental irrigation was applied to all plots at the main growth stages of pea and maize. The same irrigation rate was applied to all treatments at each irrigation event.

### Measurement and calculation

#### N accumulation

**Figure 2:**
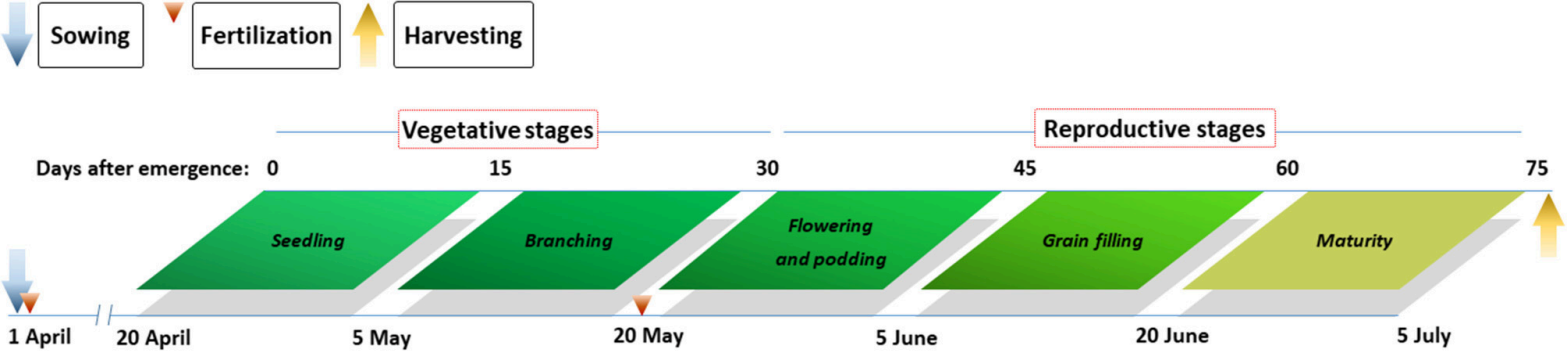
Main growth stages of pea and agronomic practices at the Oasis Agricultural Experimental Station in northwestern China in 2012–2014.

Pea samples were collected 15 d after seedling emergence, with subsequent sampling at 15-d intervals until plants achieved physiological maturity (Figure 2). At each sampling date, the entire aboveground portion was harvested from all pea plants in four 30-cm long adjacent rows in sole and intercropping systems to assess aboveground dry matter. Among the harvested samples, leaves, stems, pods, and grains were separated and oven dried at 105°C for the first 30 min and subsequently at 80°C until a constant mass was achieved. After oven drying, the entire portion of each sample was ground to a fine powder, passed through a 1-mm sieve, milled, and mixed thoroughly for the analysis of N content. Tissue N content was measured by dry combustion using an Elementar vario MACRO cube (Elementar, Hanau, Hessen, Germany). N accumulation (Nac) in tissues (kg N ha^−1^) was calculated as the product of N content and the corresponding dry matter. To fit the plant growth with N accumulation, the following logistic growth equation was used:

$$\begin{array}{l} Y=\frac{A}{1+e^{B-kt}} \end{array}$$

where, *Y* is the aboveground N accumulation at *t* day, *A* is the maximal accumulated N, and *B* and *k* are the fit parameters.

#### N allocation

N accumulation in different tissues of pea plants varies during development (Salon et al., 2001). Therefore, the proportion of N accumulated in each plant tissue, defined as nitrogen allocation (NA), is vital for assessing the N status of a given species, and was calculated as:

$$\begin{array}{l} NA_{i}\;\left(\%\right)=\frac{{Nac}_{i}}{{Nac}_{t}}\times \;100 \end{array}$$

where, NA_i_ and *Nac*_*i*_ represent N allocation and accumulation in the *i*th plant tissue, respectively, and *Nac*_*t*_ represents the total N accumulation in plants, defined as the sum of N accumulation in each plant tissue. Different plant tissues include leaves, stems, and grains; pods were treated as stems in this study because of similar N concentrations in both tissues.

#### N translocation

During reproductive stages of pea development, some of the N accumulated in vegetative organs is translocated to grains. Thus, a cropping system that is efficient in transferring N from vegetative organs to grains is effective in harvesting N. The quantity and rate of N translocation from vegetative organs to grains, and the contribution of transferred N to grain development were determined with the following equations:

$$\begin{array}{l} NTQ=LNa-FNa\\ NTR\left(\%\right)=\frac{NTQ}{LNa}\times \;100\\ NTC\left(\%\right)=\frac{NTQ}{{Nac}_{g}}\times \;100 \end{array}$$

where, NTQ (kg N ha^−1^) and NTR are the quantity and rate of transferred N, respectively; *LNa* and *FNa* are the largest and final amounts of N accumulated in vegetative organs, respectively; NTC is the contribution of N to grains transferred from vegetative organs; and *Nac*_*g*_ is N accumulation in grains.

The N translocation advantage of intercropping (NTA), defined as the difference in N contribution between intercropping and sole cropping, was estimated based on the NTC of intercropped pea relative to that of sole pea, according to the following equation:

$$\begin{array}{l} NTA=\frac{{NTC}_{I}}{{NTC}_{S}} \end{array}$$

where, *NTC*_*I*_ is the NTC of intercropped pea, and *NTC*_*S*_ is the NTC of sole pea. A value of NTA >1.0 indicates an advantage of intercropped pea in N translocation, < 1.0 indicates a disadvantage, and equal to 1.0 indicates no difference between intercropped and sole-cropped pea.

#### Symbiotic N fixation

At the harvest of pea crop, pea and maize plants were sampled for δ^15^N analysis, and the δ^15^N of pea (δ^15^N_pea_), and non-N-fixing maize (δ^15^N_maize_) were determined via mass spectrometry (DELTAplus XP, Thermo Finnigan, Bremen, Germany). The percent N derived from the atmosphere (%Ndfa) was determined using the ^15^N content of pea and maize plants, according to the following equation (Shearer and Kohl, 1986):

$$\begin{array}{l} \%Ndfa=\frac{\delta^{15}N_{maize}-\delta^{15}N_{pea}}{\delta^{15}N_{maize}-B}\times \;100 \end{array}$$

where, δ^15^N_maize_ and δ^15^N_pea_ are the δ^15^N value of maize and pea, respectively, and *B* = −2.05 for the pea cultivar used in the current study (Hu et al., 2017). The present research confirmed the use of the natural abundance method, as (i) δ^15^N of field soil in the 0–20 cm layer was 7.32%0 and (ii) analysis precision was ±0.2%0 (Peoples et al., 2002). The amount of N fixed (Ndfa) was calculated as the product of pea biomass, %N content, and %Ndfa. The ratio of Ndfa to total N accumulation (Ndfa/TN) was also calculated.

#### NutE

Per-unit N accumulation that contributed to grain yield was defined as NutE (g biomass g^−1^ N), and was calculated as follows:

$$\begin{array}{l} NutE=\frac{GY}{{Nac}_{t}} \end{array}$$

where, *GY* is the grain yield of pea, and *Nac*_*t*_ is the total N accumulated in pea plants.

### Statistical analysis

Statistically significant differences among treatments was examined using one-way analysis of variance (ANOVA) for a split plot factorial design with SPSS 17.0 (SPSS Institute Inc.), where plots were considered as random effects, while year, cropping system, and N management system were considered as fixed effects. Comparison of means was performed using the least significant difference (LSD) test, and differences were declared significant at *P* < 0.05. Because year × treatment interaction was significant for most dependent variables in this study, treatment effects were assessed for each year individually, unless otherwise stated. Linear associations between dependent variables were evaluated using Pearson's correlation coefficient.

## Results

### N accumulation characteristics

#### N content

**Figure 3:**
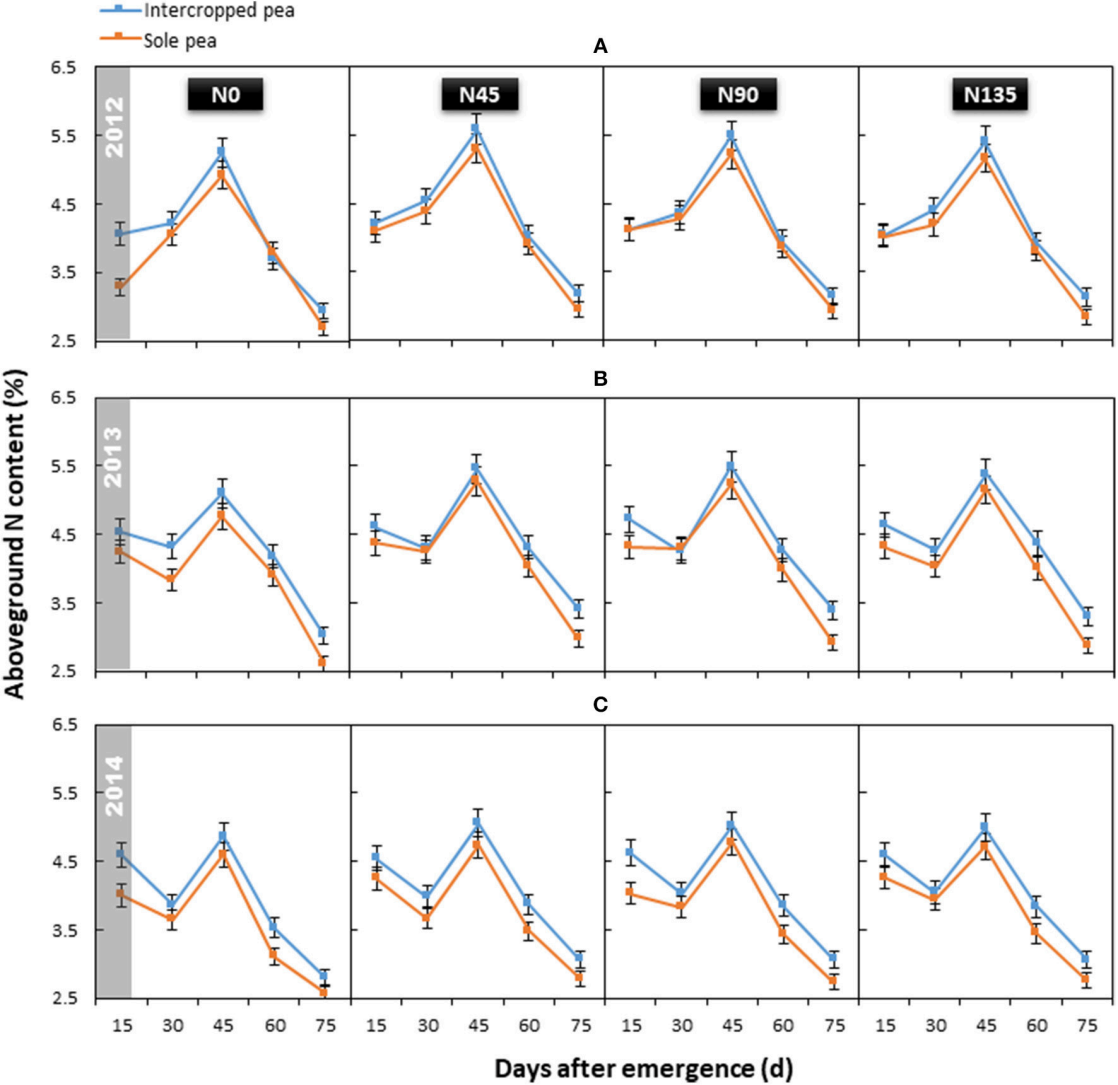
Aboveground N content of intercropped pea and sole pea in **(A)** 2012, **(B)** 2013, and **(C)** 2014. N0 is the non-N fertilized control. N45, N90, and N135 represent N fertilizer applied at 90 kg N ha^−1^ as base fertilizer at sowing plus 45, 90, and 135 kg N ha^−1^ top-dressed at flowering, respectively. Error bars are standard errors of the means.

The year × treatment interaction did not significantly affect average aboveground N content of pea (*P* = 0.964); however, aboveground N content differed significantly among treatments and sampling dates, and this effect varied with year. Generally, N content declined from initial branching (15 d after emergence) to flowering (30 d after emergence), except in 2012. Then, the N content increased and reached a peak (mean = 5.12%) at initial grain filling (45 d after emergence), and subsequently declined until maturity (Figure 3).

Cropping system (*P* ≤ 0.024), N fertilization treatment (*P* < 0.001) and cropping system × N management system interaction (*P* ≤ 0.042) significantly affected aboveground N content of pea. In intercropped pea, N45 treatment produced the highest average aboveground N content (4.28%), which was on an average 5.2% greater than that of the control (N0; 4.06%) across all sampling times (Figure 3). In the sole pea crop, the average aboveground N content with N45 treatment was improved by 8.0% compared with N0 treatment (4.03% in N45 vs. 3.73% in N0). In both cropping systems, no significant differences were observed between the average aboveground N content among treatments receiving N fertilizer (N45, N90, and N135). Additionally, intercropping consistently improved the aboveground N content of pea throughout the study period (2012–2014) by an average of 6.9% compared with the sole pea crop.

#### N accumulation

**Figure 4:**
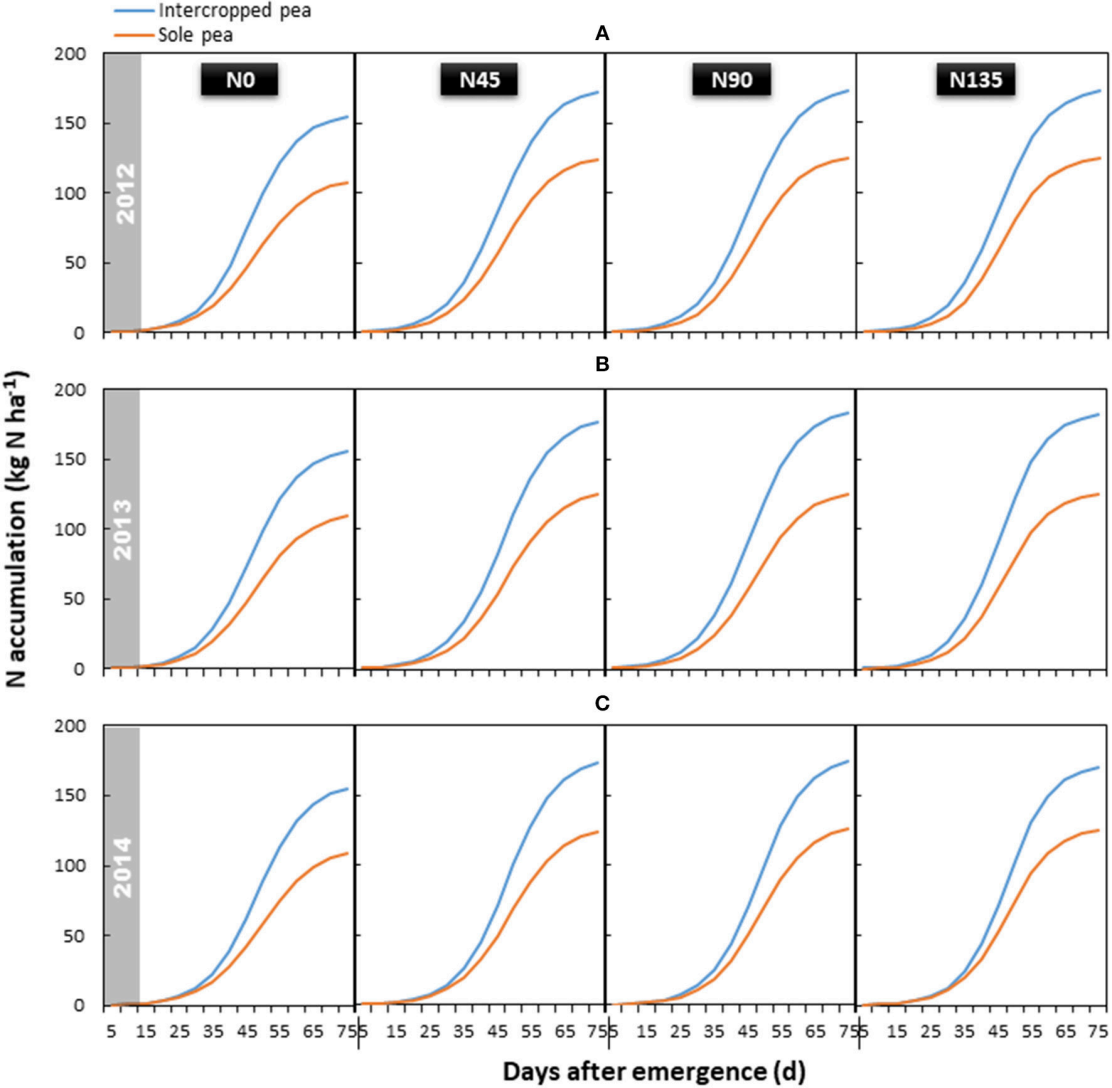
Aboveground N accumulation of intercropped pea relative to sole pea in **(A)** 2012, **(B)** 2013, and **(C)** 2014. N0 is the non-N fertilized control. N45, N90, and N135 represent N fertilizer applied at 90 kg N ha^−1^ as base fertilizer at sowing plus 45, 90, and 135 kg N ha^−1^ top-dressed at flowering, respectively.

Total aboveground N accumulation followed a similar trend in each year, and was significantly influenced by the year × treatment interaction (*P* < 0.001) (Figure 4). Compared with sole cropping, intercropping increased total N accumulation in pea by 39.6, 43.9, and 38.6% in 2012, 2013, and 2014, respectively. The N135 treatment resulted in the highest accumulation of total N, which was 13.3, 16.0, and 11.7% higher than that in the N0 treatment in 2012, 2013, and 2014, respectively; however, no significant differences were observed among the three treatments receiving N fertilizer.

#### N allocation

The allocation of N to leaf, stem, and pod tissues of pea plants was significantly affected by the cropping system and N fertilization treatment during reproductive stages (45–75 d after seedling emergence) and the effect varied with plant tissues and sampling dates (Table 2). With continued plant growth, N allocation continuously decreased in leaves but consistently increased in pods through the vegetative and reproductive stages; however, in stems, N allocation increased during vegetative growth and decreased during reproductive growth.

**Table 2:** Nitrogen allocation (%) among leaf, stem, and pod fractions of pea at 15, 30, 45, 60, and 75 d after emergence as affected by cropping system and N fertilization treatment, averaged across 2012–2014.

**Cropping system^a^**	**N treatment^b^**		**15**		**30**		**45**			**60**			**75**		
			**Leaf**	**Stem**	**Leaf**	**Stem**	**Leaf**	**Stem**	**Pod**	**Leaf**	**Stem**	**Pod**	**Leaf**	**Stem**	**Pod**
	Intercropped	N0	55.1	44.9	50.6	49.4	37.9	34.6	27.5	24.7	38.6	36.7	6.3	21.7	72.0
	Pea	N45	**55.4^c^**	**44.6**	**49.1**	**50.9**	35.7	46.5	17.8	25.0	51.6	23.4	5.0	23.1	71.8
		N90					32.7	41.5	25.8	22.6	45.2	32.2	6.5	25.0	68.6
		N135					30.1	37.1	32.8	23.0	45.3	31.6	8.0	24.1	67.9
	Sole pea	N0	54.8	45.2	51.0	49.0	39.8	43.6	16.6	26.4	39.0	34.6	7.0	21.9	71.2
		N45	**55.1**	**44.9**	**48.6**	**51.4**	36.5	49.8	13.7	28.5	51.3	20.2	5.1	22.9	71.9
		N90					35.5	43.8	20.7	27.0	45.4	27.6	7.7	25.1	67.2
		N135					33.0	47.2	19.7	26.7	47.4	25.8	8.6	26.2	65.3
	LSD (0.05)^d^		−	−	−	−	2.4	2.3	2.0	0.2	4.0	0.9	0.5	0.5	1.3
*P > F*															
	Cropping system (C)	NS	NS	NS	NS	0.037	0.000	0.000	0.000	0.038	0.000	0.004	0.013	0.028	
	N treatment (N)	NS	NS	NS	NS	0.000	0.000	0.000	0.019	0.000	0.000	0.000	0.000	0.000	

a *Intercrop and sole crop means the intercropped pea and sole pea*.

b *N0 is the non-N fertilized control. N45, N90, and N135 represent N fertilizer applied at 90 kg N ha^−1^ as base fertilizer at sowing plus 45, 90, and 135 kg N ha^−1^ top-dressed at flowering, respectively*.

c *Data in bold font represent the averaged value for N1, N2, and N3, as N fertilization did not differ among these treatments at 15 and 30 d after emergence*.

d *LSD was for the treatments in the same column*.

Averaged across years and N fertilizer treatments, intercropping significantly decreased N allocation in leaf tissues of pea plants by 5.8, 12.2, and 9.2% at 45, 60, and 75 d after emergence, respectively, compared with sole cropping (Table 2). Averaged across years and cropping systems, N allocation to leaf tissues at 45 and 60 d after pea emergence was highest in the N45 treatment, which was 5.9 and 7.9% higher than that with the N90 treatment, respectively, and 14.4% and 7.6% higher than that with the N135 treatment, respectively. At 75 d after pea emergence, N allocation to leaf tissues in the N45 treatment was 28.9% and 39.2% lower than that in N90 and N135 treatments, respectively.

The allocation of N to stem tissues of intercropped pea plants at 45 and 60 d after emergence in the N45 treatment was on an average 12.0 and 14.2% higher than that in the N90 treatment, respectively, and 25.3 and 13.9% higher than that in the N135 treatment, respectively (Table 2). Similarly, the N45 treatment increased N allocation to stem tissues of sole-cropped pea by 13.7 and 13.0% compared with the N90 treatment, respectively, and by 5.5 and 8.2% compared with the N135 treatment, respectively. Averaged across cropping systems, N allocation to stem tissues in the N45 treatment at 75 d after pea emergence was reduced by 8.2 and 8.5% compared with the N90 and N135 treatments, respectively. At 45 and 60 d after emergence, N allocation to pod tissues of intercropped pea plants in the N45 treatment was on an average 31.0 and 27.3% lower than that in the N90 treatment, respectively, and 45.7 and 25.9% lower than that in the N135 treatment, respectively. In sole-cropped pea, N allocation to pod tissues at 45 and 60 d after emergence was lower in the N45 treatment by an average of 33.8 and 26.8% compared with N90, respectively, and by 30.5 and 21.7% compared with N135, respectively. Across cropping systems, N allocation to pod tissues at 75 d after pea emergence was 5.8 and 7.9% higher in the N45 treatment compared with N90 and N135 treatments, respectively.

### N translocation

Across years, the NTQ and NTC for pea leaf and pod tissues were significantly affected by cropping systems and N fertilization treatments, whereas NTR was significantly affected only by N fertilization treatments (Table 3). In addition, the interaction between cropping system and N fertilization treatment significantly affected NTQ for pea leaf and pod tissues (Table 3). In general, values of NTQ, NTR, and NTC were higher in intercropping than in sole cropping, and the N45 treatment showed the highest values of NTQ, NTR, and NTC among all four N fertilization treatments.

**Table 3:** Quantity of transferred N (NTQ), rate of transferred N (NTR), contribution of N in vegetative organ transferred to grain (NTC), and N translocation advantage of intercropping (NTA) as affected by cropping system and N fertilization treatment, averaged across 2012-2014.

**Cropping system^a^**	**N treatment^b^**	**Leaf**				**Stem**			
		**NTQ (kg)**	**NTR (%)**	**NTC (%)**	**NTA**	**NTQ (kg)**	**NTR (%)**	**NTC (%)**	**NTA**
Intercropped	N0	0.046	77.4	33.7	1.20	0.047	52.1	34.6	1.13
pea	N45	0.080	84.6	39.6	1.26	0.106	62.0	52.0	1.10
	N90	0.064	76.0	36.5	1.24	0.076	53.6	43.3	1.04
	N135	0.058	71.7	33.4	1.22	0.074	51.5	42.6	1.02
Sole pea	N0	0.033	76.3	28.2	-	0.035	47.5	27.4	-
	N45	0.052	81.9	31.8	-	0.078	59.6	47.7	-
	N90	0.046	75.9	29.6	-	0.065	53.2	41.5	-
	N135	0.041	70.9	28.5	-	0.062	54.3	42.2	-
LSD (0.05)^c^		0.003	0.14	0.03	0.02	0.004	0.23	0.03	0.03
*P > F*									
Cropping system (C)		0.000	NS^d^	0.000	-	0.000	NS	0.017	-
N treatment (N)		0.000	0.000	0.009	0.005	0.000	0.000	0.000	0.003
C × N		0.002	NS	NS	-	0.004	NS	NS	-

a *Intercrop and sole crop means the intercropped pea and sole pea*.

b *N0 is the non-N fertilized control. N45, N90, and N135 represent N fertilizer applied at 90 kg N ha^−1^ as base fertilizer at sowing plus 45, 90, and 135 kg N ha^−1^ top-dressed at flowering, respectively*.

c *LSD was for the treatments in the same column*.

d *NS refers to no significant differences between treatments at 0.05 levels*.

In intercropped pea, the NTQ for leaf in N45 treatment was 25.0 and 37.9% higher than that in N90 and N135 treatments, respectively, while that for stem was 39.5 and 43.2% higher, respectively (Table 3). In sole-cropped pea, NTQ for leaf in N45 treatment was 13.0 and 26.8% higher than N90 and N135 treatments, respectively, and that for stem was 20.0 and 25.3% higher, respectively. Averaged across cropping systems, N45 treatment increased the NTR for leaf and stem by 9.6 and 13.9%, respectively, compared with N90 treatment, and by 14.8 and 14.9%, respectively, compared with N135 treatment.

The NTC in pea for leaf and stem was higher in intercropping by 21.3 and 8.1%, respectively, compared with sole cropping. Among treatments, the NTC for leaf and stem in N45 treatment was higher by 8.0 and 17.6%, respectively, than N90, and by 15.3 and 17.6%, respectively, than N135 (Table 3). The N45 treatment also increased the NTA for both leaf and stem tissues. The NTA for leaf and stem in N45 treatment was 1.6 and 5.8% higher than N90, respectively, and 3.3 and 7.8% higher than N135, respectively.

### Symbiotic N fixation

During the 3-year study period, cropping system significantly affected Ndfa and Ndfa/TN, and N fertilization treatment significantly affected %Ndfa, Ndfa, and Ndfa/TN (Table 4). Generally, intercropping increased Ndfa but decreased Ndfa/TN compared with sole cropping, and the N45 treatment consistently improved %Ndfa, Ndfa, and Ndfa/TN compared with N90 and N135 treatments. The value of %Ndfa was higher in N45 treatment by 22.2, 12.6, and 16.6% in 2012, 2013, and 2014, respectively compared with N90, and by 33.5, 45.1, and 17.8% in 2012, 2013, and 2014, respectively, compared with N135. Intercropping increased the value of Ndfa by an average of 34.4% compared with sole cropping over the three years. In addition, the N45 treatment improved Ndfa by 21.8, 9.6, and 17.2% in 2012, 2013, and 2014, respectively, compared with N90, and by 37.1, 46.3, and 20.6% in 2012, 2013, and 2014, respectively, compared with N135. Additionally, N45 improved Ndfa/TN by 21.6, 12.0, and 18.5% in 2012, 2013, and 2014, respectively, compared with N90, and by 35.9, 48.6, and 19.9% in 2012, 2013, and 2014, respectively, compared with N135.

**Table 4:** Percent nitrogen derived from air (%Ndfa), amount of nitrogen derived from air (Ndfa, kg N ha^−1^), and the ratio of Ndfa to total N accumulation (Ndfa/TN, %) in 2012-2014 as affected by cropping system and N fertilization treatment.

**Treatment**	**%Ndfa**			**Ndfa**			**Ndfa/TN**		
	**2012**	**2013**	**2014**	**2012**	**2013**	**2014**	**2012**	**2013**	**2014**
**CROPPING SYSTEM EFFECT (C)^a^**									
Intercrop	39.5	47.0	53.0	64.2	73.3	86.8	38.5	42.4	51.9
Sole crop	41.5	50.3	54.3	48.0	54.4	64.5	40.2	45.2	53.5
*P*-value	0.407	0.585	0.099	0.012	0.002	0.001	0.406	0.517	0.601
LSD (0.05)	7.1	7.3	5.3	12.6	9.3	9.1	9.3	7.0	7.0
**N TREATMENT EFFECT (N)^b^**									
N0	49.8	59.1	61.8	63.5	71.2	80.8	48.6	54.0	61.2
N45	43.7	52.6	56.4	63.0	70.9	82.7	42.6	47.2	55.8
N90	35.8	46.7	48.4	51.8	64.7	70.6	35.0	42.2	47.1
N135	32.7	36.3	47.9	46.0	48.5	68.6	31.3	31.8	46.5
*P*-value	0.006	0.004	0.000	0.010	0.001	0.008	0.000	0.000	0.000
LSD(0.05)^c^	7.3	5.8	5.4	8.8	7.8	7.1	5.5	4.9	5.4
C × N	NS^d^	NS	NS	NS	NS	NS	NS	NS	NS

a *Intercrop and sole crop means the intercropped pea and sole pea*.

b *N0 is the non-N fertilized control. N45, N90, and N135 represent N fertilizer applied at 90 kg N ha^−1^ as base fertilizer at sowing plus 45, 90, and 135 kg N ha^−1^ top-dressed at flowering, respectively*.

c *LSD was for the treatments in the same column*.

d *NS refers to no significant differences between treatments at 0.05 levels*.

### Grain N removal

#### NutE

**Figure 5:**
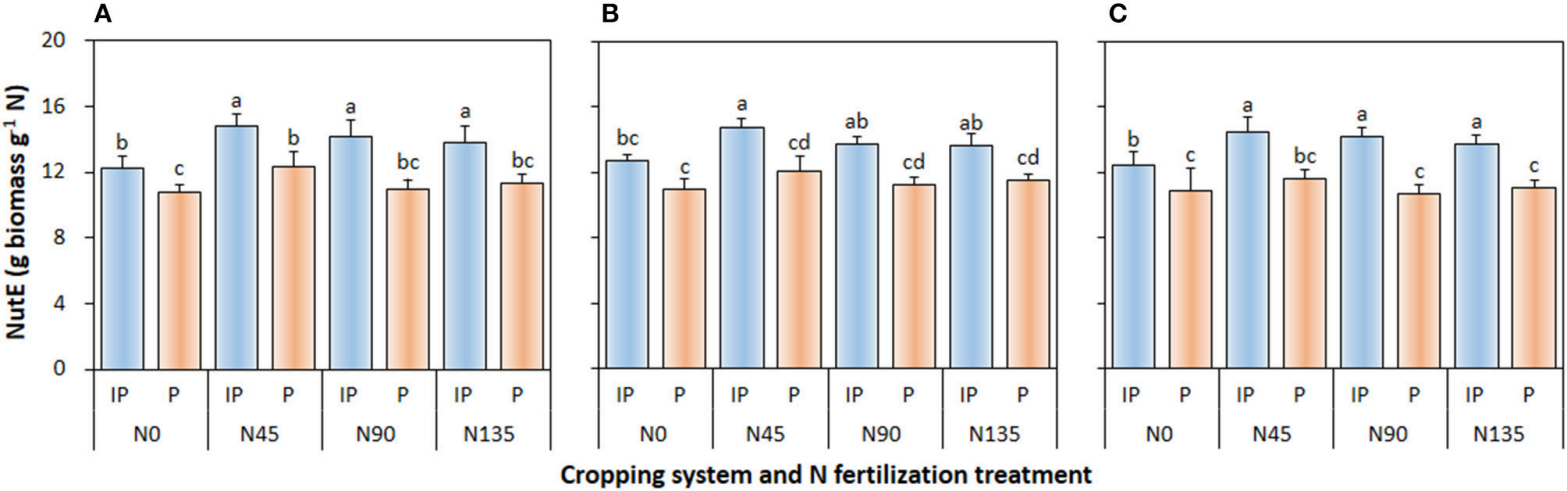
Nitrogen utilization efficiency (NutE) of intercropped pea relative to sole pea in **(A)** 2012, **(B)** 2013, and **(C)** 2014. IP, intercropped pea; P, sole pea. N0 is the non-N fertilized control. N45, N90, and N135 represent N fertilizer applied at 90 kg N ha^−1^ as base fertilizer at sowing plus 45, 90, and 135 kg N ha^−1^ top-dressed at flowering, respectively. Error bars are standard errors of the means.

Cropping system (*P* ≤ 0.002), N management system (*P* < 0.001), and cropping system × N fertilization treatment interaction (*P* ≤ 0.006) significantly affected NutE of pea in all three study years (Figure 5). In intercropped pea, the N45 treatment produced the highest NutE, which was 4.6 and 7.9% higher than that in N90 treatment in 2012 and 2013, respectively, and was 6.9, 8.2, and 5.7% higher than that in N135 in 2012, 2013, and 2014, respectively. Similarly, in sole-cropped pea, N45 produced the highest NutE, which was 12.4, 7.1, and 9.1% higher than that in N90 in 2012, 2013, and 2014, respectively, and 9.2, 5.0, and 4.9% higher than that in N135 in 2012, 2013, and 2014, respectively. Moreover, compared with sole cropping, intercropping consistently improved NutE of pea crop by an average of 20.9, 19.9, and 24.0% in 2012, 2013, and 2014, respectively.

#### NHI

**Figure 6:**
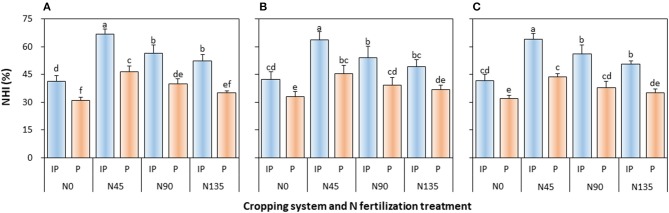
Nitrogen harvest index (NHI) of intercropped pea relative to sole pea in **(A)** 2012, **(B)** 2013, and **(C)** 2014. IP, intercropped pea; P, sole pea. N0 is the non-N fertilized control. N45, N90, and N135 represent N fertilizer applied at 90 kg N ha^−1^ as base fertilizer at sowing plus 45, 90, and 135 kg N ha^−1^ top-dressed at flowering, respectively. Error bars are standard errors of the means.

The NHI of pea was significantly affected by the cropping system (*P* ≤ 0.003), N fertilization treatment (*P* < 0.001), and cropping system × N fertilization treatment interaction (*P* ≤ 0.001) in all study years (Figure 6). In intercropped pea, NHI was the highest in N45 treatment, and was higher by 18.4, 18.2, and 13.7% than that in N90 treatment in 2012, 2013, and 2014, respectively, and by 27.9, 29.3, and 26.7% than that in N135 treatment in 2012, 2013, and 2014, respectively. In sole cropping, N45 treatment also produced the highest NHI, which was 17.0, 15.2, and 15.4% higher than that in N90 treatment in 2012, 2013, and 2014, respectively, and 32.3, 22.5, and 24.4% higher than that in N135 treatment in 2012, 2013, and 2014, respectively. In addition, intercropping consistently enhanced NHI on an average by 42.1, 35.4, and 42.8% in 2012, 2013, and 2014, respectively, compared with the sole pea crop.

### Relationship of Na-t, NutE, and NHI with NTR, NTC, and Ndfa

**Figure 7:**
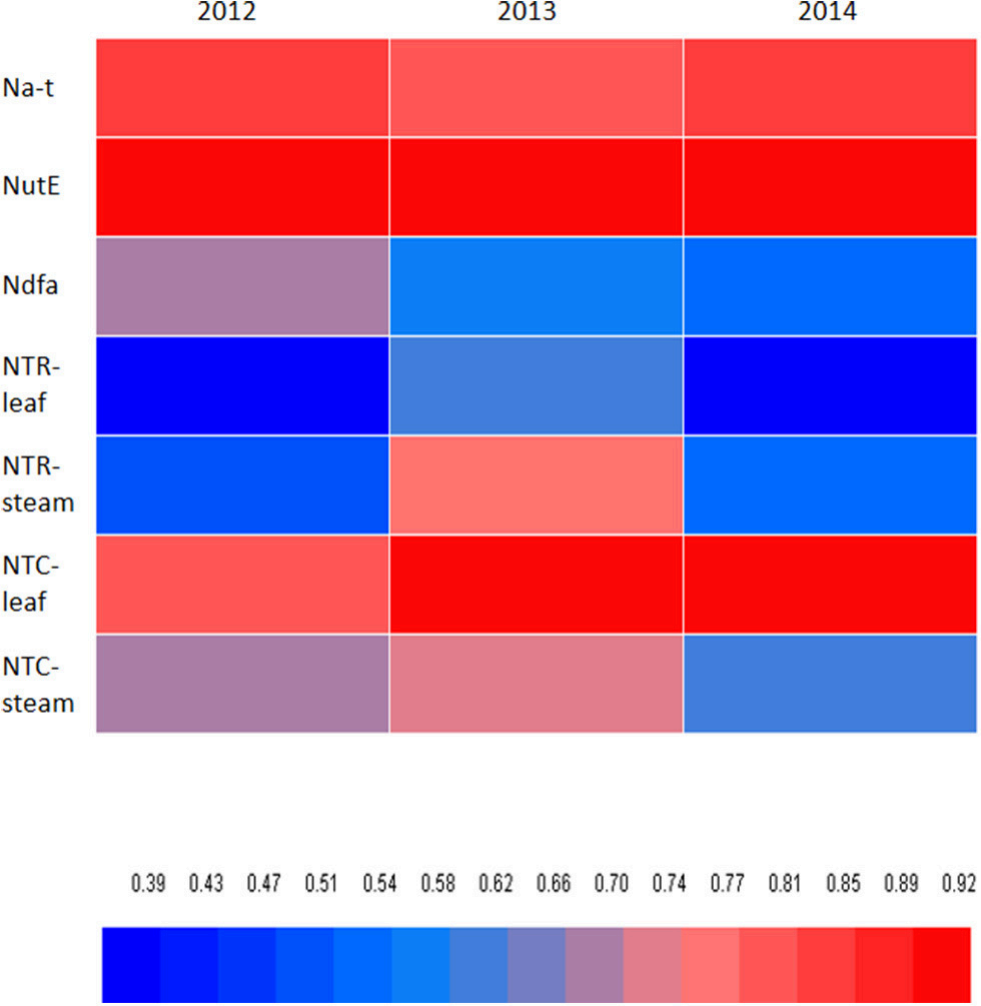
Pearson's correlation coefficient of nitrogen harvest index (NHI) relative to total N accumulated in pea plants (Na-t), nitrogen utilization efficiency (NutE), amount of nitrogen derived from atmosphere (Ndfa), rate of transferred N (NTR) of leaf and steam, and contribution of nitrogen in vegetative organ transferred to grain (NTC) of leaf and steam. Blue color means low correlation, and read color means high correlation.

A significant positive correlation of NTR for stem (*P* = 0.027) and NTC for leaf (*P* < 0.001) and stem (*P* < 0.001) was observed with Na-t (Table 5). These findings indicate that N translocation from vegetative organs to grains is vital for the final N accumulation in pea; this final N accumulation is more closely associated with N translocation from stem than from leaf. The significant positive correlation of NutE with NTR and NTQ for leaf (*P* ≤ 0.032) and stem (*P* ≤ 0.011) indicates that N translocation substantially facilitates grain formation. A significant positive linear association (*P* = 0.003) was also observed between Ndfa and NutE, implying that biological N_2_ fixation can potentially improve the translocation of accumulated N to grain. Additionally, NHI showed a positive correlation with NTR and NTC of leaf (*P* ≤ 0.019) and stem (*P* ≤ 0.001), Ndfa (*P* = 0.014), and NutE (*P* < 0.001) (Figure 7). This indicates that increasing N translocation from vegetative organs to grains or enhancing N_2_ fixation in pea will greatly improve N harvesting in grains.

**Table 5:** Pearson's correlation coefficient and the corresponding statistical significance of total N accumulated in pea plants (Na-t), nitrogen utilization efficiency (NutE), and nitrogen harvest index (NHI) relative to rate of transferred N (NTR), contribution of nitrogen in vegetative organ transferred to grain (NTC), and amount of nitrogen derived from atmosphere (Ndfa) across 2012–2014.

**Variable**	**Na-t**	**NutE**	**NHI**
NTR-leaf	0.100^a^	0.522^**^	0.474^*^
NTR-stem	0.450^*^	0.517^**^	0.626^**^
NTC-leaf	0.734^**^	0.440^*^	0.848^**^
NTC-stem	0.702^**^	0.830^**^	0.725^**^
Ndfa	0.284	0.579^**^	0.496^*^
NutE	−	−	0.953^**^

a Correlation coefficients followed by ^*^ and ^**^ are significant at P ≤ 0.05 and P ≤ 0.01, respectively.

## Discussion

### Effect of cropping systems

N is an essential element required for crop growth (Kaur et al., 2017). In cropping systems, N fertilization is often needed to economically optimize crop yield (Zhang et al., 2011). The rate and time of N fertilizer application, along with its placement and source, are vital factors affecting the management practices for improving NutE (IFA, 2009). In the present study, N fertilizer application was divided into two portions, with 90 kg N ha^−1^ applied at sowing and the remaining amount (45, 90, or 135 kg N ha^−1^) top-dressed at flowering. Throughout the study period (2012–2014), the rate of N fertilizer application at top-dressing significantly influenced the N status of pea plants, and integration of intercropping substantially facilitated this effect on N content, translocation, and NHI.

The approach of integrating N fertilizer management with intercropping to maximize NutE and crop productivity is supported by previous research. In China, a lower N fertilization rate integrated with wheat–cotton (*Gossypium hirsutum* L.) intercropping enhanced agronomic N use efficiency; however, N management strategies with sole-cropping of wheat and cotton resulted in high N surplus, thus increasing environmental risk (Zhang et al., 2008). In Italy, reducing the rate of fertilizer N application and integrating durum wheat (*Triticum durum* Desf.)-field bean (*Vicia faba* L.) intercropping have been shown to improve forage yield with better quality; whereas, sole-cropping of durum wheat and field bean with higher N fertilization rate reduces N uptake (Mariotti et al., 2012). In Denmark, reducing the rate of N fertilization by 50% along with wheat–pea intercropping has been shown to increase N yield and grain N concentration; however, sole-cropping of wheat and pea with 80 kg N ha^−1^ increases N input, resulting in negative environmental impact (Ghaley et al., 2005). Consistent with these studies, our results showed that pea–maize intercropping increased N concentration in pea grains compared with sole cropping and traditional N fertilizer application.

#### Intercropping enhances the effect of N fertilization

Generally, intercropped components increase NutE because of differences in (i) genotype and physiological metabolism, (ii) time, rate, and amount of N uptake, (iii) canopy configuration, and (iv) rooting system (Lithourgidis et al., 2011a; Furseth et al., 2012). This advantage of intercropping helps optimize N acquisition and distribution in plant tissues, thus increasing the NHI, especially in grain sinks (Ghaley et al., 2005). In this study, the content of N in aboveground tissues of pea plants was enhanced by N fertilizer management and cropping system. In the N45 treatment, sole cropping was more effective than intercropping at increasing aboveground N content, as the increase in average aboveground N content in N45 treatment compared with N0 treatment was 8.0% in sole cropping and 5.2% in intercropping. However, the increase in the absolute value of average aboveground N content in N45 treatment compared with N0 was ≥4.06% in intercropping and ≤ 4.03% in sole cropping. Thus, intercropping enhanced the effect of N fertilizer on increasing N content in aboveground tissues of pea plants. Consequently, total N accumulation of intercropped pea in N45 treatment was increased by 39.2% compared with sole pea in N135 treatment. However, the aboveground N content and Na-t in pea did not differ significantly among the three treatments receiving N fertilizer; thus, the N45 treatment was the most efficient. This might be attributable to intensified interspecific competition under low soil N conditions, leading pea plants to absorb a greater proportion of soil-derived N than N applied as fertilizer (Bedoussac and Justes, 2011; Hu et al., 2017).

In this study, N allocation was more variable in leaf and pod tissues than in stems, indicating that leaves and pods are more important organs for N storage and transport (Hay et al., 1953; Dreccer et al., 2000). At 15 d after emergence, more N was allocated to leaves than to stems; however, at flowering, N was almost equally allocated to leaves and stems. This effect has been attributed to greater transport capacity for export of N from leaves, combined with a reduction in N storage capacity of leaves (Salon et al., 2001), followed by leaf senescence (Schjoerring et al., 1995). In the present study, N allocation to leaves decreased from 35.2 to 6.8%, while that in pods increased from 21.8 to 69.5%. These data support N translocation from leaves to grains (Ogawa et al., 2016). Moreover, N fertilization significantly affected N allocation to plant organs (Ceh-Breznik and Tajnšek, 2005; Jiang et al., 2005), and the effect varied with the growth stage. With the decreasing rate of N top-dressed at flowering, N allocation to leaves increased at 45 and 60 d after pea emergence but decreased at physiological maturity (75 d after pea emergence). These results imply greater potential for N translocation from leaves at lower N fertilization rate, which is consistent with previous studies (Mu et al., 2016; Ogawa et al., 2016). Intercropping intensified the effect of N fertilization rate on enhancing the N translocation capacity of leaf and N storage capacity of pod. This was evident from the decreased N allocation to leaves and increased N allocation to pods in intercropped pea compared with sole-cropped pea in the N45 treatment.

In this study, N transportation capacity was quantified as the quantity and rate of N translocation (Xu et al., 2006; Shi et al., 2007). The amount of N translocation from stems was greater than that from leaves, whereas N translocation rate from stems was less than that from leaves. This might be attributed to several reasons. First, the translocation of N is easier from leaf than from stem because of less storage capacity of leaves at senescence (Mu et al., 2016; Ogawa et al., 2016). Second, N in leaf was translocated to stem prior to pod, thereby increasing the amount of N transferred from stem. Third, the absolute value of the difference between the largest and final N accumulation was greater in stem than in leaf. A lower rate of N fertilizer (i.e., 45 kg N ha^−1^) at flowering enhanced the quantity and rate of N transferred from both leaf and stem, which was further facilitated by intercropping. Compared with N90 and N135 treatments, the N45 treatment increased NTQ from leaf by 25.0 and 37.9%, respectively, and NTQ from stem by 39.5 and 43.2%, respectively, with intercropping; in sole-cropped pea, N45 increased NTQ from leaf by 13.0 and 26.8%, respectively, and NTQ from stem by 20.0 and 25.3%, respectively. Accordingly, NTC was significantly higher in N45 treatment compared with N90 and N135 treatments, and in intercropping compared with sole cropping. Thus, N45 treatment improved the NTA from leaf and stem 5.8 and 7.8%, respectively, compared with N135 treatment.

Fixation of atmospheric N2 in legume crops is vital for maximizing crop yield (Peoples et al., 2002). However, N_2_ fixation is strongly inhibited by abiotic factors such as soil inorganic N content (Hardarson et al., 1984). Numerous studies have evaluated strategies to alleviate the inhibitory effect of soil inorganic N on N_2_ fixation, and have identified that reducing N fertilization is the most effective approach for preventing the inhibition of N_2_ fixation (Li et al., 2009; Hu et al., 2017). In the present study, N_2_ fixation by pea plants was greater in the N45 treatment than in N90 and N135 treatments. The N45 treatment increased %Ndfa, Ndfa, and Ndfa/TN on an average by 17.1, 16.2, and 17.4%, respectively, compared with N90, and by 32.1, 34.7, and 34.8%, respectively, compared with N135. In addition, intercropping facilitated N_2_ fixation of pea on an average by 34% across N fertilization treatments, compared with sole-cropping. The approach of intercropping legumes with cereals for maximizing symbiotic N_2_ fixation is widely adopted in arid regions (Li et al., 2009). Higher N_2_ fixation with intercropping has mainly been attributed to interspecific interactions (Corre-Hellou et al., 2006; Hu et al., 2016). In addition intercropping could also enhance the effect of N fertilization on N_2_ fixation. The average Ndfa values were 82.9 and 61.1 kg N ha^−1^ in N45 and N135 treatments, respectively, in intercropping, 61.5 and 47.5 kg N ha^−1^, respectively, in sole cropping. This corresponds to 36% and 30% improvement in Ndfa in N45 treatment compared with N135 treatment for intercropped and sole-cropped pea plants, respectively.

N affects grain yield (Mae, 1997); thus, increases in both N uptake and NutE are vital for improving grain yield (Samonte et al., 2006; Nasri et al., 2014). In the present study, the N45 treatment, with improved Na-t and NutE, resulted in higher grain yield compared with N90 and N135 treatments in both intercropping and sole cropping systems. Correlation analysis indicates that improved Na-t and NutE are fundamentally attributable to enhanced translocation of N from both leaf and stem. Moreover, the contribution of N translocation was more important for stem than for leaf, consistent with the study of Schjoerring et al. (1995). Biological N_2_ fixation also appeared to facilitate NutE, as indicated by the significant positive linear association between Ndfa and NutE. This may be because fixed N acts as a source of storage compounds that support N accumulation in grains (Salon et al., 2001). Our results also revealed that intercropping intensified the N fertilizer effect on NutE, as intercropping increased (by 34 and 30%) the difference of NutE with N45 compared to N90 and N135. A significant positive correlation was also observed between NutE and NHI, implying that increasing N translocation is effective in boosting N concentration in grain sinks, or in other words, improving N remobilization (Kairudin and Frey, 1990; Schjoerring et al., 1995). Accordingly, the N45 treatment, with greater quantity and rate of N translocation compared with N90 and N135 treatments, also showed the highest NHI. Integration with intercropping bolstered the effect of N45 treatment on the improvement in NHI. This is demonstrated by the increase in NHI in the N45 treatment by 66% compared with N90 treatment, and by 82% compared with N135 treatment for intercropped pea vs. sole-cropped pea.

Our results clearly show that integrating optimal N fertilizer management for pea (i.e., 90 kg N ha^−1^ applied at planting plus 45 kg N ha^−1^ top-dressed at flowering) with intercropping (i.e., pea–maize strip intercropping) is an effective cropping model for sustainable agriculture, as this combination can (i) reduce synthetic N fertilizer input, (ii) enhance biological N_2_ fixation, (iii) improve N remobilization efficiency, and (iv) increase N concentration in grain sinks. To adopt this approach, it is important to take into account that pea is a (i) cool-season crop planted ~20 d earlier than maize; (ii) dominant species, which strongly competes against maize (Hu et al., 2016); and iii) leguminous crop capable of fixing atmospheric N, thereby offsetting N deficiency in soil. To establish this system as a sustainable cropping model, further research is needed on N use efficiency, especially on apparent N recovery, and N balance including nitrous oxide emission, ammonium volatilization, and nitrate leaching.

## Conclusion

Overall, we showed that N fertilizer management in pea with 90 kg N ha^−1^ at sowing and 45 kg N ha^−1^ top-dressed at flowering (N45 treatment), integrated with maize–pea intercropping, was efficient at increasing the NHI of pea plants. This integrated system resulted in similar quantity of N accumulation with less N fertilization compared with the N135 treatment (90 kg N ha^−1^ at sowing and 135 kg N ha^−1^ top-dressed at flowering), while significantly improving NTQ, NTC, and NTA. Additionally, this system achieved the highest N_2_ fixation, NutE, and NHI, and revealed a significant positive correlation among N_2_ fixation, NutE, and NHI, indicating that enhancing N_2_ fixation can greatly improve the N concentration in pea grain. Thus, intercropping pulses with less N fertilization will not only improve the protein content but also decrease the potential loss of available soil N. To comprehensively evaluate this integrated farming system, further studies are needed to determine the effect of this system on grain nutritional quality, soil residual N, and economic benefits.

## Author contributions

QC conceived the idea and led the study design. FH and YT carried out the experiment, performed analysis and wrote the paper. AY, CZ, and JC critically reviewed the manuscript. ZF, WY, and HF assisted with study design and experiments. All authors approved the final version of the manuscript.

### Conflict of interest statement

The authors declare that the research was conducted in the absence of any commercial or financial relationships that could be construed as a potential conflict of interest.
